# COP27: The Prospects and Challenges for the Middle East and North Africa (MENA)

**DOI:** 10.34172/ijhpm.2022.7800

**Published:** 2022-11-05

**Authors:** Amirhossein Takian, Arefeh Mousavi, Martin McKee, Vahid Yazdi-Feyzabadi, Ronald Labonté, Viroj Tangcharoensathien, Ruairí Brugha, Elizabeth Bradley, Lawrence Gostin, Eivind Engebretsen, Nir Eyal, Sharon Friel, Victor G. Rodwin, Ole F. Norheim, Mohammad Hajizadeh, Naoki Ikegami, Agnes Binagwaho, Ilona Kickbusch, Aidin Aryankhesal, Ali-Akbar Haghdoost

**Affiliations:** ^1^Department of Global Health & Public Policy, School of Public Health, Tehran University of Medical Sciences, Tehran, Iran.; ^2^Department Health Management, Policy & Economics, School of Public Health, Tehran University of Medical Sciences, Tehran, Iran.; ^3^Health Equity Research Center (HERC), Tehran University of Medical Sciences, Tehran, Iran.; ^4^Health Management and Economics Research Center, Isfahan University of Medical Sciences, Isfahan, Iran.; ^5^Centre for Global Chronic Conditions, London School of Hygiene and Tropical Medicine, London, UK.; ^6^Health Services Management Research Center, Institute for Futures Studies in Health Kerman University of Medical Sciences, Kerman, Iran.; ^7^School of Epidemiology, Public Health and Preventive Medicine, University of Ottawa, Ottawa, ON, Canada.; ^8^Ministry of Public Health, Nonthaburi, Thailand.; ^9^Department of Public Health and Epidemiology, Royal College of Surgeons in Ireland, Dublin 2, Ireland.; ^10^Vassar College, Poughkeepsie, NY, USA.; ^11^O’Neill Institute for National and Global Health Law, Georgetown University Law Center, Washington, DC, USA.; ^12^Faculty of Medicine, University of Oslo, Oslo, Norway.; ^13^School of Public Health, Rutgers, The State University of New Jersey, New Brunswick, NJ, USA.; ^14^Center for Population-Level Bioethics, Rutgers University, New Brunswick, NJ, USA.; ^15^Menzies Centre for Health Governance, The Australian National University, Canberra, ACT, Australia.; ^16^Wagner School of Public Service, New York University, New York City, NY, USA.; ^17^Department of Global Public Health and Primary Care, University of Bergen, Bergen, Norway.; ^18^School of Health Administration, Dalhousie University, Halifax, NS, Canada.; ^19^Keio University, Tokyo, Japan.; ^20^University of Global Health Equity, Kigali, Rwanda.; ^21^Graduate Institute for International and Development Studies, Geneva, Switzerland.; ^22^Iran University of Medical Sciences, Tehran, Iran.; ^23^Research Centre for Modelling in Health, Institute for Future Studies in Health, Kerman University of Medical Sciences, Kerman, Iran.

**Keywords:** COP27, Climate Change, MENA Region, Health Effects

## Abstract

In line with the global trend, the Middle East and North Africa (MENA) region has been growing vulnerable to the direct and indirect health effects of climate change including death tolls due to climatological disasters and diseases sensitive to climate change since the industrial revolution. Regarding the limited capacity of MENA countries to adapt and respond to these effects, and also after relative failures of the previous negotiation in Glasgow, in the upcoming COP27 in Egypt, the heads of the region’s parties are determined to take advantage of the opportunity to host MENA to mitigate and prevent the worst effects of climate change. This would be achieved through mobilizing international partners to support climate resilience, a major economic transformation, and put health policy and management in a strategic position to contribute to thinking and action on these pressing matters, at least to avoid or minimize the future adverse consequences.

## Introduction

 The latest report of the United Nations Intergovernmental Panel on Climate Change (IPCC) warned that the Earth is warming faster than previously thought and called it a “code red for humanity.”^1^ Although the definition of climate change does not match the classical definition of epidemics, its impacts on human race extinction are far greater than the Coronavirus disease 2019 (COVID-19) devastating pandemic.^2^ Thus, climate change is considered the “biggest global health threat of the 21st century” and its repercussions hinder the achievement of the Sustainable Development Goals.^3^ Despite the threat climate change poses to all of human life, its impacts are uneven in various geographical areas worldwide, while also disproportionately affecting poorer and socially marginalized populations.^4^

## Climate Change Effects on the Middle East and North Africa Region

 Being one of the hottest and driest regions, consequences from climate change is more intense throughout countries of the Middle East and North Africa (MENA) region. Even without drought, water shortage limits economic and social development in most countries in MENA; many countries focus on coping with acute water scarcity rather than drought per se.^5^ The possibility of increasing the risk of death from extreme temperature and heat wave in the distant future (2070–2099) for RCP 8.5 scenario is estimated to increase 8 to 20 fold over current risks if no climate change mitigation is implemented.^4^ The trend of increasing temperature in many parts of MENA is unbearable, especially for the elderly, women, children, and outdoor workers in particular immigrant construction workers. With agriculture as the largest sector in terms of its water consumption (82%), over 60% of MENA citizens live in the areas of water-stress, against the 35% global average.^6^ Desertification, changes in rainfall patterns, and rising sea levels in coastal areas have exposed MENA to various climatological hazards, especially drought, dust, and floods. Taken together, these developments mean that the region is severely exposed to many adverse health effects of climate change, such as food insecurity, child malnutrition, and vector-borne diseases, eg, Zika and dengue fever,^7,8^ among others.

## The MENA Region’s Vulnerability

 MENA has been experiencing rapid population growth (1.7% in 2021),^9^ urbanization (66% in 2021),^10^ population displacement, dependency on food imports, inadequate access to clean water, and inequitable access to healthcare and education.^11^ Exacerbated by the devastating consequences of the COVID-19 pandemic, long-term conflicts and war, complex humanitarian emergencies, and the ongoing economic stagnation, as 11 out of 17 MENA economies may not recover to pre-pandemic levels by the end of 2022,^12^ the capacity of MENA countries to adapt and respond to the direct and indirect effects of climate change is limited.^13^ It has been estimated that climate change accounted for 0.4% to 1.3% gross domestic product decrease in the MENA countries, with concern that this could rise to 14% gross domestic product reduction if mitigation and adaptation measures are not adopted.^14^

## Adaptation and Mitigation Measures

 Despite some MENA’s countries attempts to advance climate commitments and move to low-carbon and climate-resilient economics, the need for more progressive and global actions is abundantly clear.^15^ As the oil industry is still the major driver of economy in many countries in the region,^16^ the significant dependency on fossil fuel production and consumption and its growing consequences on climate change are not sustainable. In 2019, eight MENA member states were among the eleven countries with the highest greenhouse gases (GHG) emissions worldwide.^17^ Hence, the urgent need to reconcile the contradictory role of the region in both generating and experiencing the negative impacts of GHG emission, necessitating adaptation measures in response to the health threats, particularly for the vulnerable and marginalized communities. We urge policy-makers, at the regional and global levels, to promote significant interventions to increase the region’s resilience to climate change and improve health systems’ resiliency to protect people from emerging threats. Such interventions should be based on the recommendations of the United Nations Environment Program (calling for more substantive emission pledges as COP27),^18^ and the International Energy Agency (stating unequivocally that there should be no new oil and gas field development).^18^ Achieving this necessary level of fundamental economic and social change will require new and accountable partnerships based on establishing sustainable peace^19^ and overcome political tensions in the region, while improving health systems’ resiliency to protect people from emerging threats.

## Health System Resilience

 As one of nature’s warning to us all the painful experience of the COVID-19 pandemic underscored the need to strengthen healthcare systems resilience to prepare, prevent, rapidly detect, and respond to deep disparities within and among countries in a comprehensive and sustainable manner. It is also essential to ensure that health systems’ resiliency to maintain its functions under climate pressures.^20^ The Paris Agreement marked the beginning of a new era in the global response to climate change, and although considered primarily to be global climate treaty, it is also a global public health treaty. The “right to health” in the Paris Agreement is an important call to action.^21^ The 55th resolution Regional Committee (EM/RC55/R.8) of the Eastern Mediterranean Region in 2008 provided a framework for action which emphasized the integration of health into national adaptation plans. Most Eastern Mediterranean countries, the second most climate-vulnerable World Health Organization (WHO) region after Africa, have yet to assess health vulnerability to the consequences of climate change as part of developing a national strategy for increasing their health infrastructure resilience.^2^ Managing these risks will require a focus on upstream interventions that prevent disease and promote population health. This will require multi-sectoral collaboration among health, agriculture, water, energy, transportation, and urban planning sectors, as highlighted by WHO’s Health in All Policies concept.^22^ Globally, the new pandemic instrument currently being negotiated by WHO members states also provides a major opportunity to create binding international obligations for health system capacities, as well as focusing on climate change and the right to health.

## Initiatives for Health in Glasgow COP26

 Despite some slow but insufficient progress,^23^ the COP26 recognized the importance of health in the climate negotiation in COP26. This message led to the understanding of the need for parties to create health systems resilient in the face of climate change through Health National Adaptation Plans and population vulnerability assessments. The program also focused on developing low-carbon health systems and harnessing the resulting health benefits.^24^ At the COP26 meeting, 11 of 21 MENA countries pledged to develop resilient health systems against climate change.^20^ However, the current 0.3% of the global adaptation budget to support health systems and the COP26 commitment to double the adaptation budget are far below the resource needed.^22,24^ In addition, there are legal, financial, and technological limitations for the production and use of renewable energy and the knowledge gap in the use of renewable energy, all of which hinder implementation.^25-27^ Worse still, the Glasgow Climate Agreement failed to resolve the challenge of industrialized countries’ resistance to honor their commitment to provide $100 billion in annual financing for climate actions in less developed nations.^28^ Whereas in high-income countries the main challenges are managing demand, rising costs, and decreasing healthcare overconsumption, in lower income countries the over-riding challenges remain unmet healthcare needs and applying existing tools to fulfil the promise of global health equity and universal health coverage. By allowing more of the carbon budget to be spent in lower income settings and engaging in priority setting to rapidly reduce the healthcare carbon footprint in higher income settings, greater health gains can be gained more fairly and efficiently.^29^

## COP27 Perspective in the MENA Region

 The most recent IPCC report confirmed that the window for climate action is closing rapidly.^30^ Most alarmingly, the report observed that there is no credible path to avoiding ever worsening outcomes unless the world makes dramatic changes. Following failures to meet the expectations of environmental activists and heads of states who are affected by global warming in the previous meetings, COP27 might be “the world’s last chance to prevent the worst effects of climate change.”^22^ It is crucial to design and accelerate implementation of the adaptation and mitigation measures in MENA to the best current and future match of the specific climate challenges of the region. It is expected that less-industrialized countries will pursue compensation for damages of climate change at the COP27 in November 2022 in Egypt. Importantly, the conference also aims for agreement on more ambitious targets to reduce GHG emissions, and to build the resilience of coastal communities and provide the necessary financing for renewable energy.

 Expected to be hosting both COP27 and COP28, the MENA region must seize the opportunity to mobilize international partners to support climate resilience in the region through a major economic transformation. The meeting aims to ascertain the extent to which the nationally determined contributions of MENA countries reflect a commitment to mitigation and adaptation measures. COP27 needs to be taken as an opportunity to increase health sector’s contribution in the nationally determined contributions for 2022.^22^ This will help the MENA’s oil and gas exporting countries to diversify their economies while still promoting economic growth and prosperity and reducing the waste and increase efficient use of natural resources. The latter may require a major paradigm shift and creating a macro-political and legal framework across many MENA countries to change the environmental protection procedures and issues related to climate change, targeting both the reduction of GHG emissions as well as the citizens’ vulnerability.^13^ In this regard, we urge the regional policy makers and climate activists to use the upcoming United Nations climate negotiations in MENA, hosted by the United Arab Emirates in 2023, as a transformative opportunity to galvanize the COP27 decisions for enhancing regional partnership toward identifying tailored mitigation and adaptation measures for avoiding the increasing GHG emissions, and mobilize adequate resources to protect vulnerable host communities from the most severe health effects of climate change.^14^ The upcoming COP27 in Egypt and its affiliated platforms might be the last chance for meaningful global commitment and action, not only promises, to put population health on the climate change agenda and thereby put health policy and management in a strategic position to contribute to thinking and action on these pressing matters, at least to avoid or minimize the risks of likely future consequences.

## Ethical issues

 Not applicable.

## Competing interests

 Authors declare that they have no competing interests.

## Authors’ contributions

 AHT: Conception and design, critical revision of the manuscript for important intellectual content. AM: Conception and design, drafting of the manuscript. All other authors contributed to review of the manuscript.
